# DNA replication is highly resilient and persistent under the challenge of mild replication stress

**DOI:** 10.1016/j.celrep.2022.110701

**Published:** 2022-04-19

**Authors:** Camelia Mocanu, Eleftheria Karanika, María Fernández-Casañas, Alex Herbert, Tomisin Olukoga, Mete Emir Özgürses, Kok-Lung Chan

**Affiliations:** 1 Chromosome Dynamics and Stability Group, Genome Damage and Stability Centre, University of Sussex, Brighton BN1 9RQ, UK

**Keywords:** MiDAS, G2 DNA synthesis, S-to-M DNA synthesis runover, RO3306, CDK1 inhibition, ATR-mediated replication checkpoint, mild replication stress, aphidicolin, RAD51, RAD52

## Abstract

Mitotic DNA synthesis (MiDAS) has been proposed to restart DNA synthesis during mitosis because of replication fork stalling in late interphase caused by mild replication stress (RS). Contrary to this proposal, we find that cells exposed to mild RS in fact maintain continued DNA replication throughout G2 and during G2-M transition in RAD51- and RAD52-dependent manners. Persistent DNA synthesis is necessary to resolve replication intermediates accumulated in G2 and disengage an ATR-imposed block to mitotic entry. Because of its continual nature, DNA synthesis at very late replication sites can overlap with chromosome condensation, generating the phenomenon of mitotic DNA synthesis. Unexpectedly, we find that the commonly used CDK1 inhibitor RO3306 interferes with replication to preclude detection of G2 DNA synthesis, leading to the impression of a mitosis-driven response. Our study reveals the importance of persistent DNA replication and checkpoint control to lessen the risk for severe genome under-replication under mild RS.

## Highlights

• DNA synthesis persists during G2-M transition to counteract replication stress (RS)
• RAD51/RAD52-mediated HR pathways facilitate the continuation of G2-M DNA synthesis
• Continued G2 DNA synthesis relieves RS-induced G2/M checkpoint for mitotic entry
• RO3306, but not CDK1 inhibition, non-specifically interferes with DNA synthesis

## Graphical abstract

**Figure:**
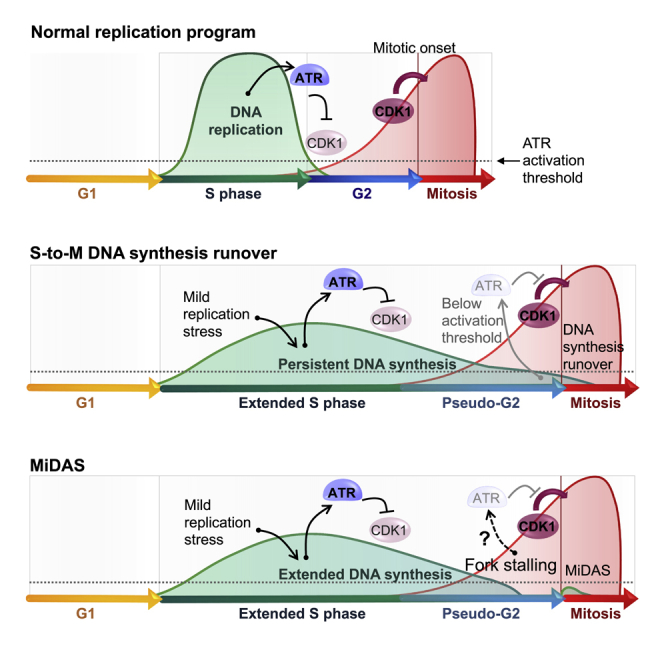

## Teaser

Contrary to the MiDAS model, in which stressed replication forks fail to proceed in late interphase unless mitosis is initiated, Mocanu et al. reveal that cells maintain continued DNA synthesis from S-phase to early mitosis to minimize genome under-replication. This response is also essential to lessen persistent ATR-mediated G2/M checkpoint activation.

## Introduction

Segregation of incompletely replicated chromosomes is a threat to genome integrity (Fernandez-Casanas and Chan, 2018; Gaillard et al., 2015). It is thus crucial to ensure that mitosis strictly follows the completion of DNA replication. DNA replication generally occurs within S phase, but under replication stress (RS) conditions, it is delayed and remains observed in G2 (Maya-Mendoza et al., 2018). Experimentally, RS can be induced by a replicative DNA polymerase inhibitor, aphidicolin (APH) (Cheng and Kuchta, 1993; Sheaff et al., 1991). At high concentrations, APH stops DNA synthesis (i.e. “strong RS”), while low doses slow replication (“mild RS”) and prolong interphase (Koundrioukoff et al., 2013; Sherwood et al., 1994). Because mild RS compromises replication completion but does not block G2-M progression (Daigh et al., 2018; Lemmens et al., 2018; Saldivar et al., 2018; Sherwood et al., 1994), it leads to chromosome fragility and sister-chromatid bridging in mitosis (Chan et al., 2009; Glover et al., 2017; Ying et al., 2013).

**Figure 1:**
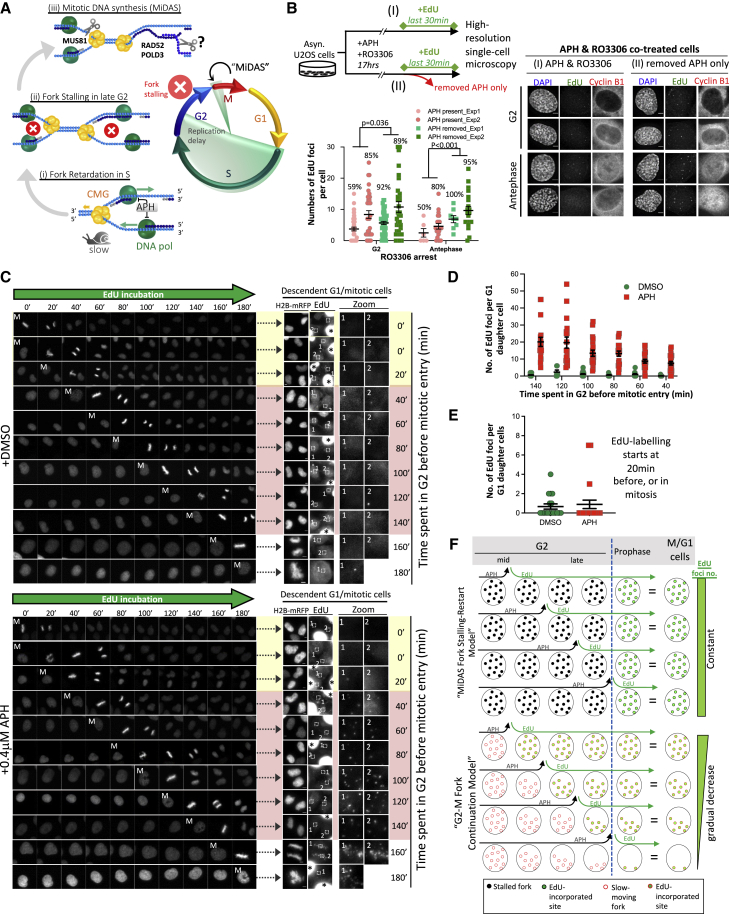
Unscheduled DNA synthesis induced by mild RS occurs before mitotic entry (A) The MiDAS model. (B) Mild RS induces DNA synthesis in G2-arrested cells. Top: experimental workflow. Right: images of G2/antephase cells with EdU foci. Bottom: quantification of EdU foci. Percentage of cells having ≥1 EdU focus, and P values are shown. (C) Time-lapse images of DMSO- and APH-treated H2B-mRFP U2OS cells undergoing mitosis (M) in the presence of EdU. Right: EdU foci in descendent cells after the 3 h movies. Asterisks show cells positive for EdU but losing H2B-mRFP. (D) Numbers of EdU foci in G1 daughter cells according to the timing of EdU addition before mitotic entry. (E) Numbers of EdU foci in G1 cells whose mother cells were incubated with EdU when in mitosis or 20 min before mitosis. (F) Models of MiDAS versus G2-M fork continuation. In the MiDAS model, replication forks stall in G2 but resume DNA synthesis after mitotic entry. Thus, the same numbers of forks are labeled irrespective of the timing of EdU incubation. In the fork continuation model, stressed forks continue throughout G2 and G2-M transition. Thus, there are more replication sites being labeled in early G2 than late G2. The numbers of EdU foci drop as cells progress to late G2 and G2-M because the completion of DNA replication. All data are represented as mean ± SEM. Scale bars, 5 μm.

A mitotic DNA synthesis pathway (MiDAS) has been proposed to activate during early mitosis to ameliorate RS-induced chromosomal instability (Minocherhomji et al., 2015). It was reported that moderately stressed replication forks fail to proceed during late interphase. In a manner strictly dependent upon mitotic entry, these inactive forks are cleaved by the structure-specific endonuclease MUS81, and resultant DNA double-stranded breaks trigger the resumption of DNA synthesis along a pathway known as break-induced replication (BIR), which requires RAD52 and POLD3 (Figure 1A) (Bhowmick et al., 2016; Costantino et al., 2014; Minocherhomji et al., 2015). Why cells would need to delay fork rescue until mitosis remains elusive. The availability of recombination-based repair pathways in G2 (Scully et al., 2019) would suggest that cells could attempt fork recovery before mitotic onset. In the same vein, how cells stabilize stalled forks and avoid triggering G2/M arrest is not fully understood (Mocanu and Chan, 2021). To address these important questions, we carefully examined DNA replication dynamics and checkpoint responses in human cycling G2 cells under mild RS. In stark contrast to the MiDAS model, we find no evidence for a cessation of DNA synthesis at moderately stressed forks, with subsequent resumption along a mitosis-specific DNA synthesis pathway. Instead, replication activity persists throughout G2 and continues into early mitosis. Perpetuated G2 replicative activity in the face of mild RS not only minimizes genome under-replication but also avoids fork stalling, attenuating the ATR-mediated replication checkpoint to promote G2-M progression. We propose that the DNA synthesis runover activity is the predominant pathway leading to mitotic DNA synthesis under mild RS. This finding contrasts with reports of MiDAS, whose detection as a mitosis-driven response may have been provoked by an off-target effect of RO3306.

## Results

### Mild RS induces unscheduled DNA synthesis before mitosis

It remains elusive what governs the suspension of replication at moderately stressed forks in late G2 (Minocherhomji et al., 2015). We thus examined DNA synthesis activity (EdU incorporation) in G2-arrested cells continuously exposed or pre-exposed to low doses of APH (Figure 1B). G2 populations were identified as cyclin B1-positive cells, with a subset showing slight chromosome condensation (Figure 1B), indicative of very late G2 phase or so-called antephase (Bullough and Johnson, 1951). Contrary to a previous study using similar conditions (Minocherhomji et al., 2015), we found many examined G2/antephase-arrested cells displaying EdU foci. Notably, APH removal further increased these EdU+ populations and the numbers of EdU foci (Figure 1B). These findings indicate that DNA replication remains active in G2 under mild RS. Moreover, replicative activity is not irreversibly subdued by low-level APH and can be restored without mitotic initiation. To further explore the ability of cells to proceed DNA synthesis in late interphase, we set up a time-lapse single-cell tracking system to determine DNA synthesis activity in naturally cycling G2 cells. Asynchronous mRFP-tagged H2B U2OS cells were pre-exposed to DMSO or APH. After drug removal, cell cycle progression was monitored in the presence of EdU for 3 h, followed by DNA synthesis analysis (Figures S1A–S1C). By matching the fixed-cell images to their corresponding time-lapse footage, we could identify original G2 cells (Figure 1C). We reasoned that if G2 and/or M phase cells were active for DNA synthesis, EdU foci should appear in G1 daughter cells. EdU foci were barely detected in G1 cells when EdU was added to mother cells in previous G2 or M phase in the DMSO controls (Figures 1C–1E). This is consistent with the completion of bulk DNA synthesis in S phase in unperturbed conditions. In sharp contrast, EdU foci were readily detected in both mitotic and G1 cells after APH treatments (Figure 1C). Retrospective timing of EdU addition to specific G2 mother cells with respect to mitosis showed that earlier EdU addition correlated with increased numbers of EdU foci in their G1 daughter cells (Figures 1C and 1D). When EdU was added to mother cells close to/during mitosis, few or no EdU foci were detected (Figure 1E). These results are incongruent with the MiDAS model, which suggests that stressed forks stall in G2, resuming DNA synthesis only after mitotic entry (or after release from RS). In this case, we would expect to see a similar number of EdU sites in G1 cells, regardless of the timing of EdU addition (Figure 1F; MiDAS model). Our finding of the gradual reduction in replication sites as cells approach mitosis implies that DNA synthesis persists until very late G2. This continued replication activity would minimize incompletely replicated sites being carried forward into mitosis (Figure 1F; G2-M fork continuation model).

### Mild RS elicits persistent DNA synthesis throughout G2 and the G2-M transition

**Figure 2:**
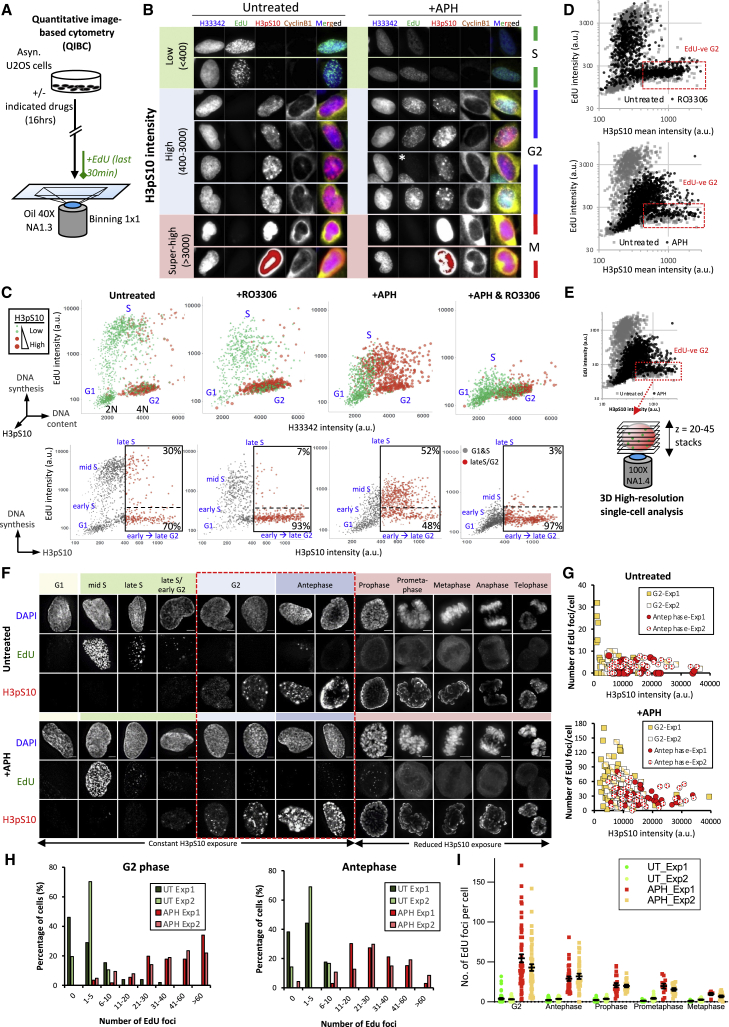
Mild replication stress leads to S-to-M DNA synthesis runover (A) Experimental workflow of QIBC. (B) QIBC images of U2OS cells treated with or without 0.4 μM APH. G2 cells show increased levels of cyclin B1 and H3pS10 phosphorylation. H3pS10 intensities were used to determine different G2 stages. An asterisk marks a G2 cell with residual EdU foci. (C) QIBC analysis. Upper panels: cell cycle distribution plots on the basis of DNA content, EdU, and H3pS10 intensities. The colors and sizes of dots represent H3pS10 intensities. Small green dots represent relatively weak H3pS10 staining, whereas large red dots show very strong signals. The threshold for G2 cells (red dots) was determined on the basis of RO3306-treated samples in which the majority of G2 cells show no EdU signals; see bottom graphs. Boxes show the percentages of G2 cells (high H3pS10) positive or negative for EdU. Early and late G2 cells were further classified on the basis of their relative H3pS10 intensities. (D) Overlays between untreated and RO3306 (upper), or APH-treated (bottom) populations. Red boxes show G2 cells gated as EdU negative. Note that a slightly higher EdU signal was detected in APH-treated populations. (E) High-resolution single-cell microscopy analysis of G2 cells classified as EdU negative in QIBC. (F) Images show DNA synthesis in different cell cycle stages of cells treated with or without APH. The acquisition condition was kept constant, except that in mitotic cells, H3pS10 exposure time was reduced to avoid saturation. G2/antephase and mitotic cells were defined on the basis of H3pS10 intensities and nuclear morphology. The red boxes showing G2 cells (classified as EdU negative in the QIBC) have EdU foci. Scale bars, 5 μm. (G) EdU foci counting as a function of H3pS10 intensity in individual G2 and antephase cells. (H) Frequencies of G2 and antephase cells with EdU foci. (I) Numbers of EdU foci per cell from G2 to metaphase. Data are represented as mean ± SEM.

The above data are consistent with the notion of replication delay after S-phase following mild RS. To address this change of replication dynamics in more detail, we used quantitative image-based cytometry (QIBC) and analyzed DNA synthesis throughout the entirety of G2 phase (Figures 2A and 2B). We used phosphorylation of histone H3 serine 10 (H3pS10) as a G2 marker because it provided excellent fluorescence dynamic ranges to distinguish early to late stages of G2 cells (Figures S1D–S1E) (Van Hooser et al., 1998). Mitotic cells were omitted in this analysis. In untreated conditions, a typical cell cycle profile was obtained with EdU signals mainly detected in the 2N-to-4N (S-phase) population characterized by low H3pS10 signals (Figure 2C, green dots). Thirty percent of H3pS10-positive cells (but with relatively low H3pS10 intensities) also showed EdU incorporation, indicative of very late S-phase populations (Figure 2C, bottom graph). As predicted, RO3306-treated samples showed G2 enrichment, displaying an increased 4N population with high H3pS10 but low EdU signals (93%) (Figure 2C, red dots; see also Figures S2A and S2B). Interestingly, upon APH treatment, the percentages of G2 cells positive for EdU nearly doubled (Figures 2C and S2B; from 30% to 52% and from 31% to 58%, respectively). Of note, EdU incorporation was also evident in cells with strong H3pS10, indicating that DNA synthesis remained present in very late G2-stage cells. However, 42%–48% of the APH-treated G2 populations were negative for EdU, raising the possibility that mild RS-induced replication delay is transitory, at least in a subset of G2 cells. Importantly, however, we noticed that the APH-treated G2 populations gated as EdU negative consistently presented with slightly higher EdU signals than control cells (Figure 2D, red boxes). On close inspection, we visualized EdU foci in these cells, while the signals are apparently not strong enough to pass the QIBC threshold (see Figure 2B, asterisk). To enhance the imaging analysis, we performed three-dimensional (3D) single-cell high-resolution microscopy and focused mainly on the G2 cells classified as EdU negative by QIBC (Figure 2E). We found that almost all the examined G2 cells under APH treatments displayed EdU foci. The number of EdU foci gradually decreased when cells had progressed through G2 and toward antephase (Figures 2F–2H). EdU foci were also detected in mitotic populations, but the numbers further dropped as cells entered and progressed through mitosis (Figure 2I). A similar result was obtained in untransformed RPE1-hTERT diploid cells (Figure S3). Together, these results strongly indicate that under mild RS conditions, DNA replication continues uninterruptedly throughout G2 phase and the G2-M transition, a phenomenon we refer to as “S-to-M DNA synthesis runover.”

### The RO3306 inhibitor, but not CDK1 inhibition per se, compromises DNA synthesis

During the QIBC analysis, we noticed that cells treated with RO3306 exhibited a significant overall drop in EdU incorporation, which was further exacerbated in the presence of APH (see Figures 2C and S2B). RO3306, which has been used in MiDAS assays to inhibit CDK1 for G2 arrest, is known to also target CDK2 *in vitro* (Vassilev et al., 2006). We reasoned that CDK2 inhibition may interfere with DNA replication *in vivo*. To investigate this, we used an engineered U2OS cell line, in which the endogenous CDK1 was replaced by the *Xenopus* CDK1 analog-sensitive (CDK1as) protein that is uniquely sensitive to the inhibition with an ATP analog, 1NM-PP1 (Rata et al., 2018). Treatment with 1NM-PP1 effectively induced G2 arrest in CDK1as cells. Importantly, unlike RO3306, 1NM-PP1 treatment did not suppress DNA synthesis activity, regardless of the presence or absence of APH (Figures S2B–S2E). Accordingly, CDK1 inhibition per se does not impair DNA replication. Vassilev and co-workers reported that RO3306 treatment delays S-phase initiation (by ∼4 h) (Vassilev et al., 2006). However, such a drastic effect was also not observed in the CDK1as G1 cells treated with 1NM-PP1 (Figure S2F). Therefore, besides mediating CDK1 inhibition, RO3306 exhibits an off-target effect that non-specifically inhibits DNA synthesis. This unwanted effect provides a plausible explanation for the lack of detection of G2 DNA synthesis reported previously (Minocherhomji et al., 2015). It follows that was what described as MiDAS in this report may have been the resumption of DNA synthesis upon RO3306 removal, which coincided with, but may have been entirely independent of, the release of cells from G2 arrest and the simultaneous removal of APH.

### RAD51 and RAD52 support continued DNA synthesis from G2 to mitosis

**Figure 3:**
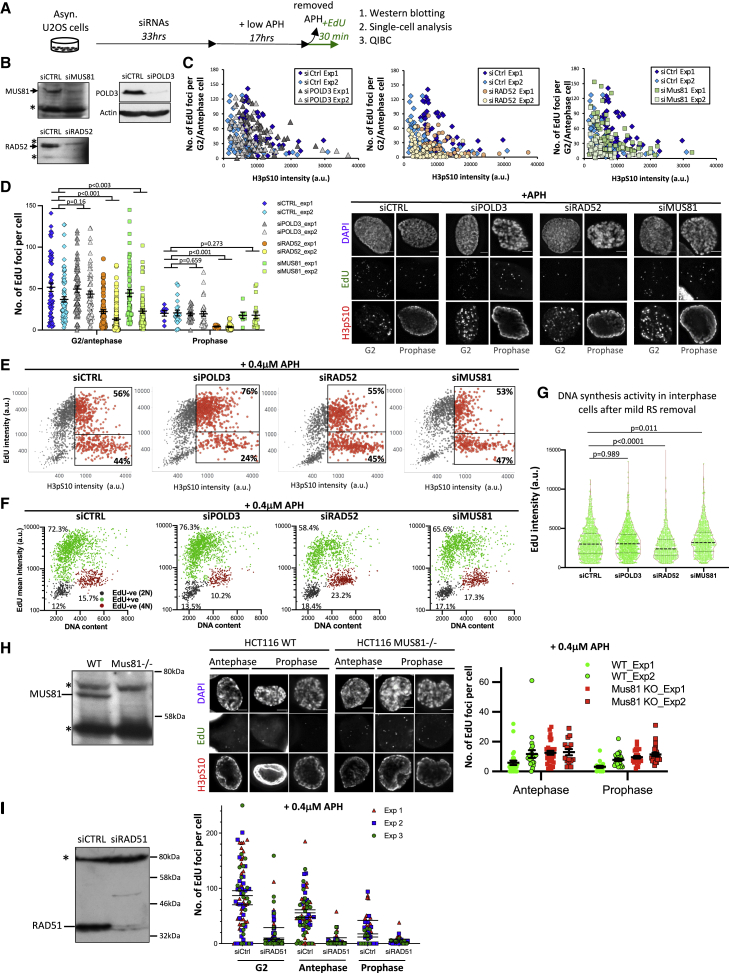
RAD52 and RAD51 depletions impair DNA synthesis in interphase and mitotic cells (A) Experimental workflow. (B) Western blotting. Asterisks, non-specific bands used as a loading control. (C) Numbers of EdU foci per cell in APH-treated G2/antephase cells according to H3pS10 intensities after RNAi. (D) Numbers of EdU foci in APH-treated G2/antephase and prophase populations. Mean ± SEM, and P values are shown. Right: representative images. Scale bars, 5 μm. (E) QIBC analysis shows EdU incorporation in APH-treated G2 populations (red dots). Boxes show the percentages of G2 cells positive and negative for EdU. The sizes of dots depict the relative H3pS10 levels. (F) Cell cycle scatterplots showing EdU signals versus DNA content. Gray dots represent G1 cells (2N), green dots show cells active in DNA synthesis, and red dots show 4N populations with low EdU signals. (G) RAD52 knockdown reduced DNA synthesis efficiency. EdU intensities of the green populations of (E). (H) Left: western blotting of wild-type (WT) and MUS81-knockout HCT116 cells. Asterisks, non-specific bands. Middle: representative images of WT and MUS81-knockout HCT116 cells having EdU foci. Scale bars, 5 μm. Right: quantification of EdU foci. Mean ± SEM is shown. (I) RAD51 depletion impairs DNA synthesis in G2, antephase, and prophase cells. Left: western blot after RAD51 RNAi. An asterisk indicates non-specific bands. Right: EdU foci counting. Means of each experiment are shown.

MUS81, POLD3, and RAD52 have been implicated in DNA synthesis during mitosis (Bhowmick et al., 2016; Minocherhomji et al., 2015). Of note, RAD52 was recently shown to be dispensable for the detection of a MiDAS phenotype in untransformed cells (Graber-Feesl et al., 2019). We wondered if these factors may involve during the S-to-M DNA synthesis runover induced by mild RS. Essentially following published MiDAS protocols (Bhowmick et al., 2016; Minocherhomji et al., 2015), but omitting the use of RO3306, we measured DNA synthesis in both cycling G2 and prophase cells after the indicated RNA interferences (Figures 3A and 3B). We found that POLD3 depletion did not cause any obvious reduction of mild RS-induced DNA synthesis in G2/antephase or prophase populations (Figures 3C and 3D). POLD3 depletion slightly increased the proportion of late S/G2 cells having active DNA synthesis (Figure 3E), which might reflect elevated replication delays. MUS81 depletion also did not cause DNA synthesis reduction in mitotic cells but a modest drop in G2/antephase populations (Figures 3C–3G). Given its purported key role in MiDAS initiation (Minocherhomji et al., 2015), we repeated our analysis in MUS81-knockout HCT116 cells. Again, we did not observe any defects in mitotic DNA synthesis (Figure 3H). These findings lead us to believe that the majority of DNA synthesis observed in mitotic prophase is a reflection of continued replication traversing from G2 interphase.

In contrast to POLD3 and MUS81, RAD52 depletion reduced EdU foci in prophase and also in G2/antephase cells (Figures 3C and 3D). It is worth noting that the drop in DNA synthesis in RAD52-depleted cells was already evident in S phase (Figures 3F and 3G). This supports a general role of RAD52 in replication fork recovery under RS (Sotiriou et al., 2016). It also implies that reduced mitotic DNA synthesis may result from gross changes in replication dynamics. Recently, another key recombination factor, RAD51, was reported to promote MiDAS (Wassing et al., 2021). Like RAD52, RAD51 functions in fork protection/recovery during interphase. Upon depletion of RAD51, EdU incorporation from G2-phase onward was much reduced (Figure 3I). We conclude that RAD51/RAD52, but not MUS81 or POLD3, are required to facilitate the continuity of G2-M DNA synthesis under mild RS. The defects of DNA synthesis observed in mitosis may simply result from the interference of the continuity of S-to-M DNA synthesis runover.

### Continued G2 DNA synthesis facilitates G2-to-M cell cycle progression

**Figure 4:**
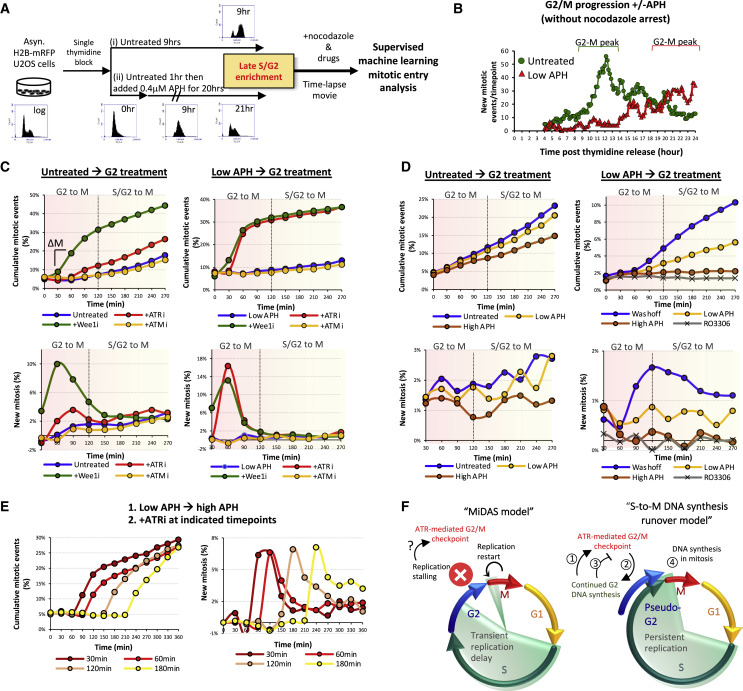
Continued DNA replication in G2 attenuates ATR-mediated G2-M arrest (A) Experimental workflow to enrich late interphase cells. Fluorescence-activated cell sorting (FACS) was performed at the indicated time points to assess cell cycle progression. (B) Live-cell analysis of mitosis in cells treated in (A). Four hours after G1/S release, time-lapse movies start without nocodazole. Increased mitotic entry was observed at 10–13 h and 19–24 h in untreated and APH-treated cells, respectively. (C) ATRi, but not ATMi, triggers instant G2-M transition in APH-pretreated G2 cells. Top: accumulative mitotic events. Bottom: net increases of mitosis per time point (ΔM). (D) DNA synthesis inhibition in pseudo-G2 (APH-pretreated) cells blocks mitotic onset. (E) ATRi rapidly bypassed G2/M arrest induced by strong RS in pseudo-G2 cells. Pseudo-G2 cells were first switched to high (4 μM) APH treatments to induce G2/M arrest, followed by ATRi treatments. (F) The S-to-M DNA synthesis runover model showing that (1) continued G2 DNA synthesis in pseudo-G2 cells maintains a constant ATR-mediated G2/M checkpoint to (2) ensure further replication completion, which subsequently (3) relieves the checkpoint brake, promoting G2-M transition but (4) causing DNA synthesis in early mitosis.

Our results indicate that cells largely carry active, ongoing (rather than stalled) replication forks into mitosis under chronic mild RS. This is interesting because replication activity has been shown to prevent mitotic initiation via an ATR-dependent checkpoint pathway (Lemmens et al., 2018; Saldivar et al., 2018). Mild RS further delays mitotic promoting factors (MPFs) expression and postpones mitotic onset (Sherwood et al., 1994). However, this checkpoint brake is not permanent. As shown here, cells eventually accumulate MPFs, entering a “pseudo-G2” stage (in which active DNA replication continues) and into mitosis. This may be because the ATR-CHK1 signaling pathway is weakly sustained by residual replication activity (Koundrioukoff et al., 2013). Besides, the MiDAS model also implies that G2 cells are competent in mitotic initiation despite fork stalling. We thus sought to examine the replication checkpoint response, particularly in pseudo-G2 cells. Using supervised machine learning, we analyzed the kinetics of mitotic entry in late interphase cells pretreated with or without APH (Figures 4A, 4B, and S4). In normal G2 populations, inhibiting Wee1 (a suppressor of CDK1), as predicted, triggered an instant G2-M transition (within 60 min). Treatment with ATMi showed no effect, and the majority of cells also did not respond to ATRi, though a small population entered mitosis earlier after 90 min (Figure 4C, left graphs). This may reflect a subset of cells under sustained ATR-dependent G2/M arrest. In sharp contrast, inhibiting ATR in pseudo-G2 populations induced a rapid surge of mitosis, similar to Wee1 inhibition (Figure 4C, right graphs), indicating broad and sustained activation of the ATR-dependent checkpoint in pseudo-G2 cells. However, as pseudo-G2 cells still proceed to mitosis, it suggests that the checkpoint activation is transient and probably relieved by continued DNA synthesis that gradually removes remaining replication structures. Supporting this, inhibiting the replication machinery in pseudo-, but not normal, G2 cells by high doses of APH elicited an instant and prolonged ATR-dependent G2/M arrest, whereas the removal of mild RS accelerated mitotic initiation (Figures 4D and 4E). Therefore, late interphase cells, if possess persistent replication structures, are constantly prevented from entering mitosis, and that the continuation of DNA synthesis activity during G2 is necessary to attenuate the ATR checkpoint and promote G2-M progression.

## Discussion

Mild RS results in DNA replication extension beyond S phase (Barr et al., 2017; Maya-Mendoza et al., 2018). The MiDAS model implied that the completion of DNA replication, for unknown reasons, is then halted in late G2, with resumption requiring a mitosis-specific event. In contrast, our findings support the view that cells drive the completion of DNA synthesis by maintaining uninterrupted DNA synthesis through to the very late stages of the cell cycle. This not only protects from potential replication shortfalls but is also a prerequisite for G2-M cell cycle progression under chronic RS. Carryover of replication structures, either in a retarded or stalled form, into G2, results in sustained ATR-mediated checkpoint signaling, blocking mitotic entry. Continued DNA synthesis with the support of recombination-based activities thus is needed to remove most replication intermediates in late interphase. Once the threshold for ATR activation is no longer reached, mitosis initiation is rapidly triggered by the accumulation of MPFs in pseudo-G2 cells. This creates an effective mechanism ensuring that most DNA synthesis is finished before mitotic initiation, but it does lead to the co-occurrence of the processes of residual DNA synthesis and chromosome condensation, generating the phenomenon of mitotic DNA synthesis (Figure 4F).

We uncover an unexpected off-target effect of RO3306 non-specifically inhibiting DNA replication. This needs to be taken into consideration when interpreting previous observations of mitotic DNA synthesis, in particular those supporting the MiDAS pathway. Studies describing MiDAS as a mitosis-driven pathway have generally applied RO3306 alongside APH (Bhowmick et al., 2016; Chappidi et al., 2020; Di Marco et al., 2017; Garribba et al., 2020; Min et al., 2019; Minocherhomji et al., 2015; Ozer et al., 2018; Sonneville et al., 2019; Wu et al., 2020). This combination of drugs largely suppresses DNA synthesis, and resumption of DNA synthesis upon drug removal may simply reflect the release of an unspecific block to DNA synthesis, not necessarily representing a mitosis-specific event. These considerations may warrant a careful reassessment of the MiDAS concept. Similarly, potential MiDAS sites have recently been mapped with protocols involving the use of RO3306 (Ji et al., 2020; Macheret et al., 2020), and the question arises whether the same sites can be identified with modified procedures avoiding this drug. Indeed, a recent study showed that treating replication-stressed cells with RO3306 can alter origin initiation and replication dynamics (Brison et al., 2020). MiDAS has also been observed at sites including telomeres and rare fragile sites (Garribba et al., 2020; Min et al., 2019; Ozer et al., 2018). Apparently, DNA synthesis can persist in prophase, but whether this merely comes from a mitosis-dependent pathway now needs further clarification. Regardless, we believe that most DNA synthesis observed in M phase under mild RS likely results from S-to-M DNA synthesis runover.

### Limitations of the study

High CDK1 activity in mitosis has been shown to trigger replisomes disassembly, leading to fork breakage and aberrant DNA end-joining in *Xenopus* cell-free extract experiments (Deng et al., 2019). Thus, replication structures, if passed into mitosis, may become unstable, and that may explain why mitotic DNA synthesis only lasts shortly (Minocherhomji et al., 2015). However, it remains plausible that some DNA synthesis detected in prophase results from DNA damage repair. However, because of the overlapping nature, distinguishing the repair reaction, if present, from the continued DNA synthesis runover is technically extremely difficult. It therefore remains a challenge to understand the biological significance of DNA synthesis during early mitosis.

## STAR★Methods

### Key resources table

**Table:** 

REAGENT or RESOURCE	SOURCE	IDENTIFIER
**Antibodies**		

Mouse anti-Cyclin B1	BD Biosciences	Cat# 610219; RRID: AB_397616
Rabbit anti-Histone H3pS10	Abcam	Cat# ab5176; RRID: AB_304763
Mouse anti-MUS81	Abcam	Cat# ab14387; RRID: AB_301167
Mouse anti-RAD52 (F-7)	Santa Cruz	Cat# sc-365341; RRID: AB_10851346
Mouse anti-POLD3	Abnova	Cat# H00010714-M01; RRID: AB_606803
Rabbit anti-RAD51	Abcam	Cat# ab63801; RRID: AB_1142428
Mouse anti-beta-ACTIN	Sigma	Cat# A5441; RRID: AB_476744
Donkey anti-mouse AF488	Invitrogen	Cat# A-21202; RRID: AB_141607
Donkey anti-mouse AF555	Invitrogen	Cat# A-31570, RRID: AB_2536180
Donkey anti-mouse AF647	Invitrogen	Cat# A-31571, RRID: AB_162542
Donkey anti-rabbit AF488	Invitrogen	Cat# A-21206, RRID: AB_2535792
Donkey anti-rabbit AF555	Invitrogen	Cat# A-31572, RRID: AB_162543
Donkey anti-rabbit AF647	Invitrogen	Cat# A-31573, RRID: AB_2536183
Rabbit Anti-Mouse HRP	Dako	Cat# P0260, RRID: AB_2636929
Donkey Anti-Rabbit HRP	ECL	Cat# NA9340-1ml, RRID: AB_772191

**Chemicals, peptides, and recombinant proteins**		

Aphidicolin (APH)	Sigma-Aldrich	Cat# A4487
VE-821 (ATRi)	Sigma-Aldrich	Cat# SML1415
KU-55933 (ATMi)	Sigma-Aldrich	Cat# SML1109
AZD1152-HQPA (Aurora Bi)	Sigma-Aldrich	Cat# SML0268
RO3306 (CDK1i)	Sigma-Aldrich	Cat# SML0569
1NM-PP1 (PP1 analog II)	Sigma-Aldrich	Cat# 529581
BI2536 (PLK1i)	Selleckchem	Cat# S1109
Neocarzinostatin	Sigma-Aldrich	Cat# N9162
Thymidine	Sigma-Aldrich	Cat# T9250
Pierce™ 16% Formaldehyde (w/v), Methanol-free	Thermo Scientific™	Cat# 28906
Vectashield	Vector Laboratories	Cat# H1000
Vectashield with DAPI	Vector Laboratories	Cat# H1200
Lipofectamine RNAi MAX	Thermo Fisher Scientific	Cat# 13778075

**Critical commercial assays**		

Click-iT™ Plus EdU Cell Proliferation Kit for Imaging, Alexa Fluor™ 488 dye	Invitrogen	Cat# C10637

**Deposited data**		

Raw counting data	This study	Mendeley Data: https://doi.org/10.17632/pntrtyfk7g.1

**Experimental models: Cell lines**		

U2OS	ATCC	Cat# HTB-96, RRID: CVCL_0042
RPE-1 hTERT	ATCC	Cat# CRL-4000, RRID: CVCL_4388
U2OS (H2B-mRFP)	This study	N/A
U2OS (CDK1as)	Dr Helfrid Hochegger, University of Sussex	PMID: 30449668
HCT116	Professor Kiyoshi Miyagawa, University of Tokyo	PMID: 16456034
HCT116 (MUS81-knockout)	Professor Kiyoshi Miyagawa, University of Tokyo	PMID: 16456034

**Oligonucleotides**		

siMUS81 CAGCCCUGGUGGAUCGAUA; CAUUAAGUGUGGGCGUCUA; UGA CCCACACGGUGCGCAA; CUCAGG AGCCCGAGUGAUA	Dharmacon	L-016143-01-0005
siPOLD3 ACGAAAACGCGUACUAAAA; GGCAUUAUGUCUAGGACUA; CAAU UAGUGGUUAGGGAAA; UGUAUA GCAAGCUGAGUAA	Dharmacon	L-026692-01-0005
siRAD52 CAGAAGGUGUGCUACAUUG; GGUCAUCGGGUAAUUAAUC; GGCC CAGAAUACAUAAGUA; GGAAGAGC CAGGACAUGAA	Dharmacon	L-011760-00-0005
siRAD51 CCACCAGACCCA GCUCCUUUAUCAA	ThermoFisher	PMID: 29445165

**Recombinant DNA**		

Plasmid mRFP-tagged H2Bj-IRES-Puro	Dr Dennis Castor, Institute of Molecular Cancer Research, University of Zurich	N/A

**Software and algorithms**		

CellProfiler Image Analysis Software	BROAD Institute; http://www.broadinstitute.org/	http://cellprofiler.org; RRID: SCR_007358
Fiji	Fiji contributors	http://fiji.sc; RRID: SCR_002285
Huygens Professional	Scientific Volume Imaging	https://svi.nl/Huygens-Professional
Ilastik	https://www.nature.com/articles/s41592-019-0582-9	https://www.ilastik.org/; RRID:SCR_015246
Spotfire	TIBCO	https://account.cloud.tibco.com/signup/spotfire; RRID: SCR_008858
Prism 9	GraphPad	https://www.graphpad.com/
ZEN 2.6 (blue edition)	Zeiss	http://www.zeiss.com/microscopy/en_us/products/microscope-software/zen.html; RRID: SCR_013672

**Other**		

Zeiss AxioObserver Z1	Zeiss	N/A

### Resource availability

#### Lead contact

Further information and requests for resources and reagents should be directed to and will be fulfilled by the lead contact, Kok-Lung Chan (koklung.chan@sussex.ac.uk).

#### Materials availability

This study did not generate new unique reagents. All stable reagents generated in this study are available from the lead contact with a completed materials transfer agreement.

### Experimental model and subject details

#### Cell culture and drug treatment

U2OS (female human osteosarcoma) and its derivatives that stably express CDK1as or H2B-mRFP cells were cultured in McCoy5A (Gibco); The H2B-mRFP U2OS cells were generated by transfection of a plasmid containing a mRFP-tagged H2Bj-IRES-puromycin cDNA. The stable cells were purified by cell sorting. HCT116 and its derivative MUS81-knockdown cells were maintained in RPMI1640 (Gibco); RPE1-hTERT (derived from female human retinal pigment epithelium) were maintained in DMEM/F12 (Sigma-Aldrich). All cell lines were regularly checked for mycoplasma and passed the mycoplasma tests (Lonza MycoAlert kit) and verified by ATCC’s cell line authentication service. All medium contains 10% fetal calf serum (FCS) and Pen/Strep antibiotics. Drug working concentrations: aphidicolin (0.4μM as a low dose; 4μM as a high dose); VE821 ATRi (4μM); KU-55933 ATMi (10μM); AZD1152-HQPA Aurora Bi (100nM); BI2536 PLK1i (100nM); RO3306 CDK1i (9μM); 1NNPP1 PP1 analog II (5μM); Neocarzinostatin (100ng/mL); Nocodazole (50ng/mL); Thymidine (2mM).

### Method details

#### Click-it chemistry and immunofluorescence assay

Drugs and EdU were added to the cultures for the indicated durations. Cells were washed with PBS once before fixation using the fixative solution (250mM HEPES pH7.4, 1xPBS, 0.1% Triton X-100, 4% methanol-free paraformaldehyde) for 20min on ice. The cells were washed five times with PBS followed by permeabilization with 0.5% of Triton X-100 in PBS for 20min on ice. After washing with PBS for five times, the samples were subjected to EdU Click-it reaction according to manufacturer’s instruction (Invitrogen). The samples were then blocked with 5% FCS in PBS for 15min before incubating with primary antibodies for 1.5hr at 37°C. The samples were washed with PBS for five times and incubated with the secondary antibodies for 30min in room temperature. For QIBC analysis, nuclei were stained with Hoechst 33,342 (0.25μg/mL) for 3min and mounted with Vectashield medium. Alternatively, they were mounted directly with Vectashield (DAPI) medium. Fluorescent images were acquired in a Zeiss AxioObserver Z1 epifluorescence microscopy system equipped with 40×/1.3 oil Plan-Apochromat, 63×/1.4 oil Plan-Apochromat and 100×/1.4 oil Plan-Apochromat objectives and a Hamamatsu ORCA-Flash4.0 LT Plus camera. In the 3D high-resolution single cell analysis, twenty-five to fifty Z-stacks were acquired at 200nm intervals. Image deconvolution was performed using Huygens Professional deconvolution software (SVI) with a measured point-spread-function (PSF) generated by 200nm-diameter TetraSpeck microspheres (ThermoFisher). Classical maximum likelihood estimation method with iterations of 40–60 and signal-to-noise of 20–60 was applied. Fiji was used to generate the representative images. Primary antibody dilution: mouse anti-Cyclin B1 (1:150), rabbit anti-Histone H3pS10 (1:400); Secondary antibody dilution: donkey anti-mouse AF488 (1:500), donkey anti-mouse AF555 (1:500), donkey anti-mouse AF647 (1:500), donkey anti-rabbit AF488 (1:500), donkey anti-rabbit AF555 (1:500), donkey anti-rabbit AF647 (1:500).

#### Live-cell tracking and EdU-incorporation analysis

H2B-RFP U2OS cells were seeded in a 2-well coverslip chamber (Sarstedt). After the addition of EdU, live-cell movies were carried out in the Zeiss AxioObserver Z1 system using a 40×/0.95 Plan-Apochromat objective. A tiling scanning of an area of 5920 μm × 2800 μm was performed every 20min for 3hr. Immediately after the movies, cells were fixed with the fixative solution. Samples were subject to EdU Click-it chemistry according to manufacturer’s instruction. A tiling scanning of the same area as in the live-cell movies was repeated but using a 63×/1.4 oil Plan-Apochromat objective. ZEN blue software was used for image and movie acquisition and for image tiles stitching. Only cells that go through a cell division or progress into mitosis were tracked and matched manually to their corresponding EdU-labelling images.

#### Quantitative image-based cytometry (QIBC)

Cells were grown on coverslips (No. 1.5) or glass-bottom 24-well plates. After the indicated treatments, they were fixed and subjected to EdU-Click-it chemistry and immunofluorescence staining as described above. Twenty-five to thirty images were acquired either under the Zeiss AxioObserver Z1 system using a 40×/1.3 oil Plan-Apochromat objective or by PerkinElmer Operetta CLS High-Content Analysis System using a 40×/1.1 water objective. Both systems use a camera with a chip size of 2048 × 2048. Images were imported into CellProfiler software for analysis. Graphs were plotted by using Spotfire or Excel software.

#### Supervised machine learning mitotic entry analysis

H2B-mRFP U2OS cells were seeded in a 4-well coverslip chamber (Sarstedt). The cells were synchronized in G1/S by a single thymidine block for 18hrs. Cells were released into fresh medium. In APH treatments, 0.4μM of APH was added 1hr post the thymidine release. Time-lapse imaging started at 9hr post the release in untreated and at 21hr in the APH-treated cells, respectively. Nocodazole (50ng/mL) was added following by a tiling scanning of a large area on both fluorescence and bright-field channels using a 20×/0.8 Plan-Apochromat objective. Images were taken every 30 min. The indicated drugs were added either before or during the movies as described in the main text. A supervised machine learning approach was adopted to count mitotic and interphase cells by using Ilastik software. A pixel and object classification module was applied to segment and identify interphase and mitotic nuclei objects. Images of random cells were chosen from different timepoints to train the deep learning neural network. Multiple correction cycles were then performed to enhance the accuracy of objection and classification prediction. A detailed procedure of mitotic cell classification was presented in Figure S4C. Some data were also manually verified for comparison (see Figure S4D).

#### Immunoblotting

Cells were trypsinized and lysed on ice for 20 min with lysis buffer (50mM Tris pH 7.5, 300mM NaCl, 5mM EDTA, 1% Triton X-100, 1.25 mM DTT, 1mM PMSF and cOmplete™ protease inhibitor cocktail). Protein concentration was quantified using a Bradford assay (Bio-Rad). Immunoblotting (IB) was performed following standard procedures. Primary antibody dilution: anti-MUS81 (1:300), anti-POLD3 (1:1000), anti-RAD52 (1:100), anti-RAD51 (1:1000) and anti-Actin (1:5000).

### Quantification and statistical analysis

Statistics analysis was performed by using GraphPad Prism version 9. Sample normality was tested by D'Agostino & Pearson, Shapiro-Wilk, and Kolmogorov-Smirnov tests. Mann-Whitney *U* test was applied according to the normality test analysis.

## Data Availability

• All raw counting data has been deposited in the Mendeley Data: https://doi.org/10.17632/pntrtyfk7g.1. • All data reported in this paper will be shared by the lead contact, Kok-Lung Chan (koklung.chan@sussex.ac.uk) upon request. • This paper does not report original code. • Any additional information required to reanalyze the data reported in this paper is available from the lead contact.

## Supplemental information

Document S1. Figures S1–S4

Document S2. Article plus Supplemental information
