# Supplementary material for: DNA replication is highly resilient and persistent under the challenge of mild replication stress
Source: Cell Rep. 2022 Apr 19;39(3):110701. doi: 10.1016/j.celrep.2022.110701 (PMC9226383; doi:10.1016/j.celrep.2022.110701)
Supplement: Document S1. Figures S1–S4 [file mmc1.pdf]

Cell Reports, Volume *39*

Supplemental information

DNA replication is highly resilient and persistent under the challenge of mild replication stress

Camelia Mocanu, Eleftheria Karanika, María Fernández-Casañas, Alex Herbert, Tomisin Olukoga, Mete Emir Özgürses, and Kok-Lung Chan

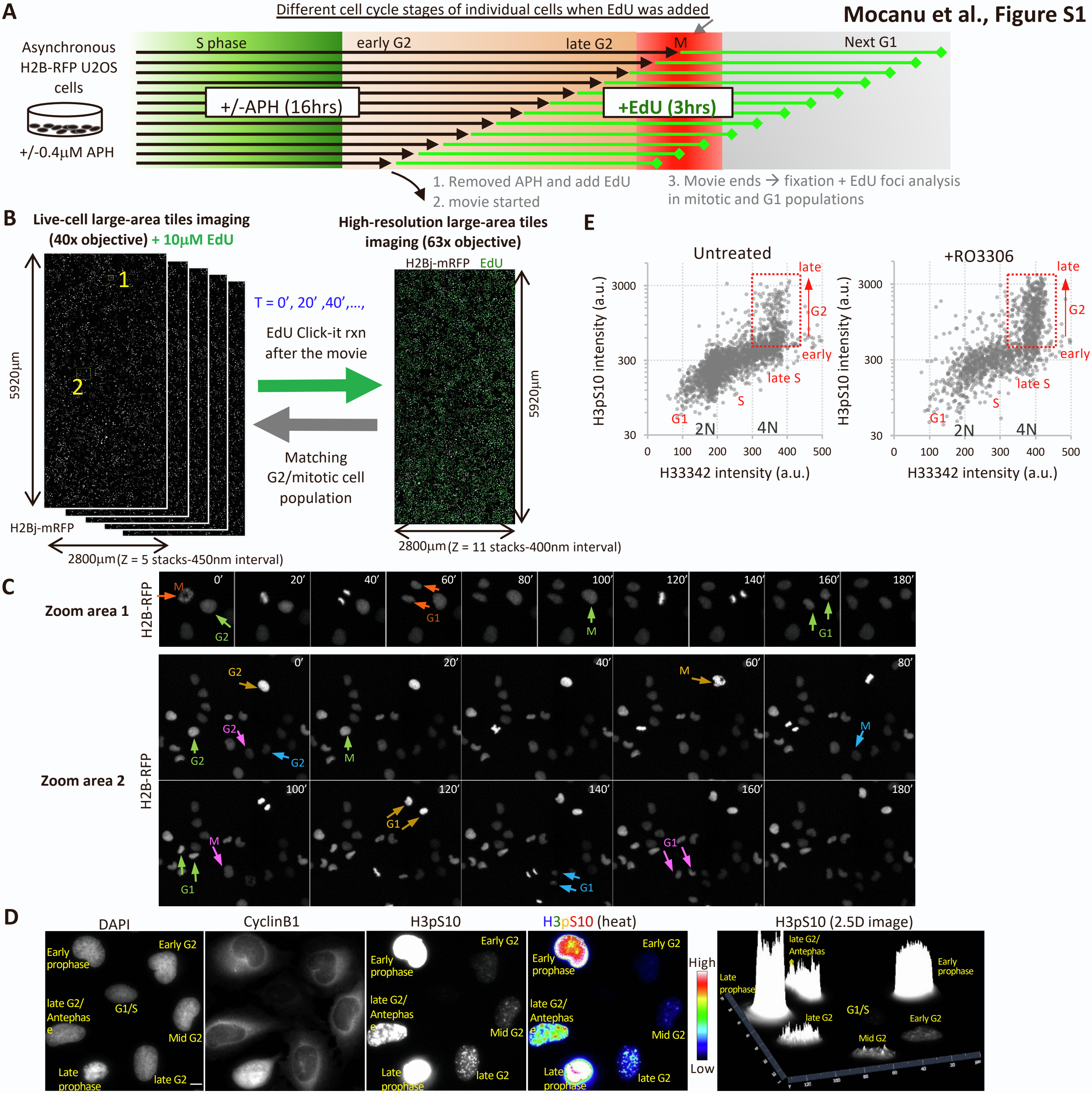

Supplementary Figure 1. Live-cell imaging and EdU labelling as a strategy for investigating DNA synthesis outside S phase (Related to Figure 1). (A) Experimental workflow of live-cell imaging combined with an EdU-based DNA synthesis assay. Asynchronous H2B-mRFP U2OS cells were untreated or treated with 0.4μM of APH for 16hrs. APH was replaced with EdU followed by time-lapse movies for 3hrs. Cells were fixed and subject to EdU click-it chemistry reaction. (B) Left: representative large-area tiling movie images of (A) captured every 20min for 3hrs. Right: a high-resolution image of the correspondent fixed cells after the movie and EdU click-it reaction. Cell were matched with the live-cell footages to analyse DNA synthesis profile of cells undergoing G2-mitosis-G1 transition. (C) Two representative examples showing the progression of G2 and M cells (single arrows) to the next G1 (double arrows). (D) Representative images of U2OS interphase cells showing the correlation between H3pS10 staining and Cyclin B1 expression. The H3pS10 staining is represented in greyscale, in a heat map and in a 2.5D image. The intensity of H3pS10 staining is used to distinguish the early, mid and late stages of G2 cells and early mitotic cells. (E) Graphs show the H3pS10 intensity plotted as a function of DNA content (H33342) in untreated and RO3306-treated U2OS interphase cells. Untreated (n=2429) and RO3306 (n=1531), where n = no. of analysed cells. Boxes denote G2 populations.

Mocanu et al., Figure S2

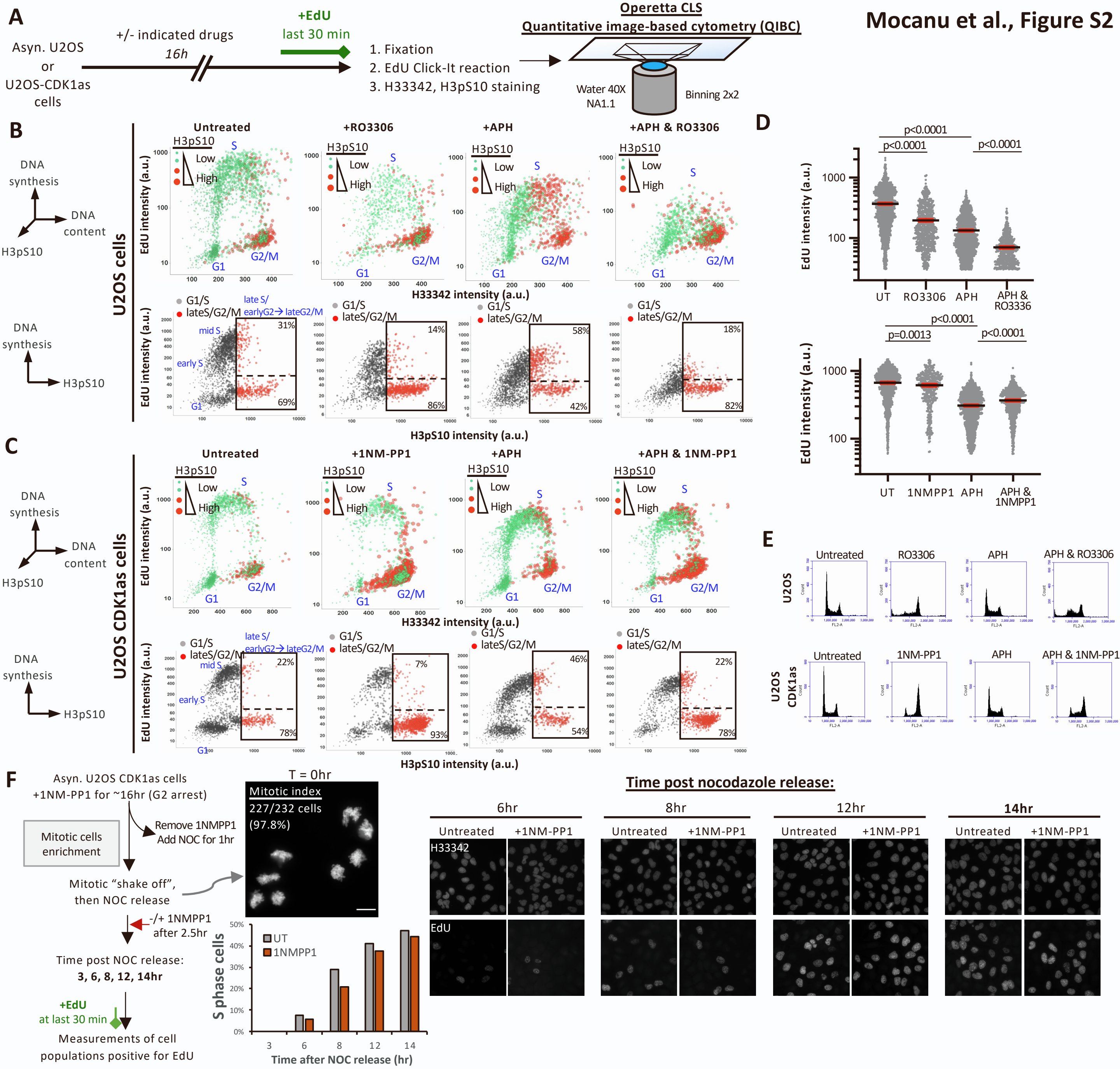

Supplementary Figure 2. RO3306, but not CDK1 inhibition, leads to non-specific suppression of DNA synthesis or severe delay in S-phase onset (Related to Figure 2). (A) QIBC using a PE Operetta CLS system equipped with a water 40x NA1.1 objective. (B) Top panels show the cell cycle profiles of U2OS cells and its CDK1as derivative under the indicated treatments. The plots were calculated based on DNA contents (H33342 intensity), EdU and H3pS10 intensities. The size and colour of the dots represent relative H3pS10 intensities. Red dots indicate cells at G2 (defined by the populations under RO3306 or 1NM-PP1 treatments, see bottom panels). Bottom panels show DNA synthesis activity versus H3pS10 intensity. Percentages of the high H3pS10 cells gated as no/low EdU and high EdU incorporation are shown. U2OS cells (untreated, n=2429; APH, n=2219; RO3306, n=1531; APH+RO3306, n=1454). (C) Same analysis as (B) but in CDK1as U2OS cells under the indicated treatments. (untreated, n=2872; APH, n=2555; 1NMPP1, n=1863; APH+1NMP11, n=1894). (D) Effects of RO3306 and 1NMPP1 on DNA synthesis activities in U2OS and CDK1as U2OS cells. Mean EdU intensities were calculated from EdU-positive populations. Mean±SEM is shown. (U2OS cells: UT, n=1552, 368.5±6.8; APH, n=1531, 133.6±2.4; RO3306, n=718, 195.9±5.9; APH+RO3306, n=565, 70.08±1.69, and U2OS-CDK1as cells: UT, n=1258, 670.2±9.0; APH, n=1375, 309.3±4.5; 1NMPP1, n=396, 615.4±16.4; APH+1NMPP1, n=789, 367.5±5.8). (E) FACS of U2OS and U2OS CDK1as cells under the indicated treatments for 16hrs. (F) CDK1 inhibition per se does not cause severe delay in S-phase initiation. The percentages of EdU positive nuclei (S-phase populations) were measured by Ilastik software. UT (T=3h, n=1322; T=6h, n=1700; T=8h, n=4191; T=12h, n=2972; T=14h, n=3165). 1NMPP1 (T=3h, n=1340; T=6h, n=3762; T=8h, n=3588; T=12h, n=2878; T=14h, n=2856). n = no. of examined cells.

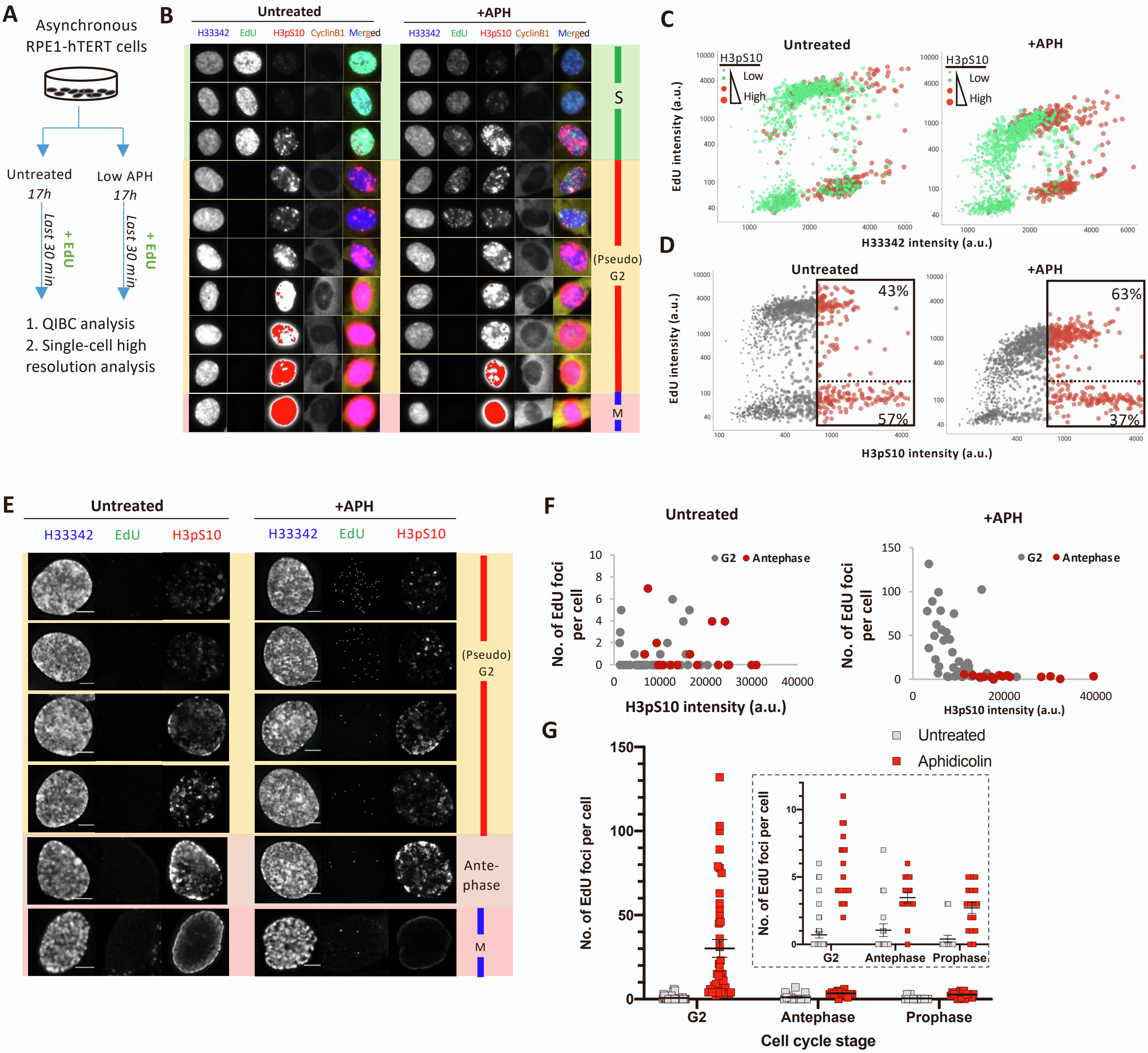

Supplementary Figure 3. RPE1-hTERT untransformed cells exhibit S-to-M DNA synthesis in response to mild replication stress (Related to Figure 2). (A) Experimental workflow. (B) Representative QIBC images of untreated and APH-treated RPE1-hTERT cells after staining of EdU, H3pS10 and CyclinB1. DNA is stained by H33342. To increase the dynamic ranges of H3pS10 imaging among the late-S and G2 cell populations, a high exposure time was employed, which lead to the signal saturation in the mitotic cells (red area). (C) Cell cycle profiles were plotted according to DNA content (H33342 mean intensity), DNA synthesis activity (EdU mean intensity) and H3pS10 mean intensities. The dot sizes and colours represent the levels of H3pS10 intensities. Untreated, n=1554 and APH, n=1498. (D) The H3pS10 intensity was plotted against the EdU mean intensity. The boxes show late S/G2 cell populations (red dots). The percentages of cells in the high H3pS10 group gated as EdU positive and negative are shown. (E) Representative images of the single-cell high-resolution imaging analysis on G2, antephase and prophase populations of hTERT-RPE1. The same imaging setting was applied to all channels between control and aphidicolin treatments. Scale bar, 5µm (F) The numbers of EdU foci in G2 and antephase cells measured by the single-cell analysis. G2 and antephase populations are classified based on the H3pS10 intensities and nuclear morphology. (G) The numbers of EdU foci of individual cells from G2 to prophase populations. The insert represents the same data set, but on a smaller Y-scale to highlight the presence of EdU foci in the antephase and prophase populations after APH treatment. Mean±SEM is shown. EdU foci in control: G2, 0.7±0.2, n=44; Antephase, 1 ± 0.5, n=18; Prophase, 0.4±0.3, n=15 and in aphidicolin: G2, 30.2±5.3, n=42; Antephase, 3.5±0.4, n=15; Prophase, 2.7±0.4, n=17. n=numbers of examined cells.

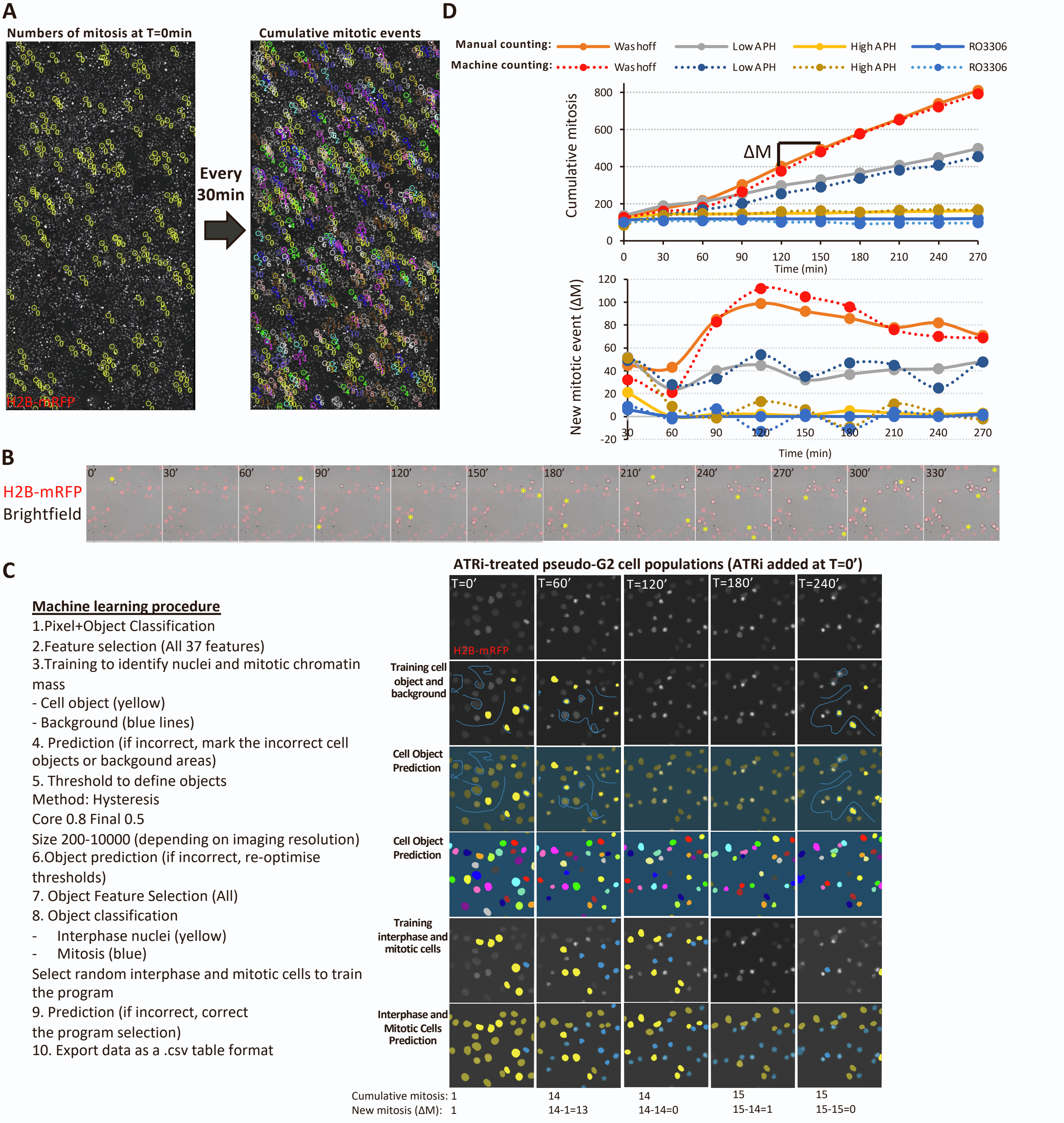

Supplementary Figure 4. Validation of supervised machine learning counting (Related to Figure 4 and STAR method). (A) Representative time-lapse live-cell images of H2B-mRFP U2OS cells. Images were taken every 30 min. Mitotic cells were manually marked (coloured circle) at each time point (numbers). (B) An enlarged region of the time-lapse images showing both H2B-mRFP and brightfield channels. Asterisks show examples of new mitotic-arrested cells at each timepoint. (C) A procedure using a part of the time-lapse images to train Ilastik software to identify and count the numbers of mitotic and interphase cells. Late S/G2 enriched H2B-mRFP U2OS cells that were cultured under a low dose of APH were treated with ATRi at T=0min. Images from random timepoints and of different areas were chosen to define background and cell objects. After a few rounds of object prediction and correction, some interphase and mitotic cells were manually chosen and used to train the software followed by mitosis/interphase cell prediction. (D) Comparison between manual and machine counting.
